# Protease-activated receptor-2: Role in asthma pathogenesis and utility as a biomarker of disease severity

**DOI:** 10.3389/fmed.2022.954990

**Published:** 2022-07-29

**Authors:** Vivek Dipak Gandhi, Nami Shrestha Palikhe, Harissios Vliagoftis

**Affiliations:** ^1^Division of Pulmonary Medicine, Department of Medicine, Faculty of Medicine and Dentistry, University of Alberta, Edmonton, AB, Canada; ^2^Alberta Respiratory Centre, University of Alberta, Edmonton, AB, Canada; ^3^School of Health Sciences and Technology, University of Petroleum and Energy Studies, Dehradun, Uttarakhand, India

**Keywords:** PAR-2, asthma, biomarker, severity, allergic disease

## Abstract

PAR_2_, a receptor activated by serine proteases, has primarily pro-inflammatory roles in the airways and may play a role in asthma pathogenesis. PAR_2_ exerts its effects in the lungs through activation of a variety of airway cells, but also activation of circulating immune cells. There is evidence that PAR_2_ expression increases in asthma and other inflammatory diseases, although the regulation of PAR_2_ expression is not fully understood. Here we review the available literature on the potential role of PAR_2_ in asthma pathogenesis and propose a model of PAR_2_-mediated development of allergic sensitization. We also propose, based on our previous work, that PAR_2_ expression on peripheral blood monocyte subsets has the potential to serve as a biomarker of asthma severity and/or control.

## Introduction

Protease-Activated Receptors (PAR) are a family of G- protein coupled receptors with 4 members, PAR_1–4_. PARs are activated by serine proteases through a unique mechanism; the extracellular N terminal of the receptor is cleaved by serine proteases and the new N terminal, the tethered ligand (TL), folds and activates the receptor (1). A variety of serine proteases produced by inflammatory and other cells or from microorganisms can activate PAR receptors (2–10). Synthetic ligands that mimic TL sequences, called PAR activating peptides (PAR-AP), can activate PAR_1_, PAR_2_, and PAR_4_ without the requirement for proteolysis. Many of the studies we will review below use PAR-AP to study PAR-mediated effects, since unlike natural proteases they do not induce PAR-independent effects. Among PARs, PAR_2_ has a wide expression pattern (11) and has been linked to inflammation in the skin (12), gastrointestinal tract (13) and lungs (14), as well as in inflammatory pain (15).

## Asthma and allergic airway inflammation

Asthma is a complex inflammatory disease of the airways and one of the most common chronic diseases worldwide (16). Based on the intensity of therapy required to maintain disease control, asthma can be classified as mild, moderate and severe (17). Severe asthma represents less than 10% of patients with asthma but is responsible for a large share of asthma associated morbidity and health care costs (18). Identification of patients with severe asthma to allow timely institution of appropriate therapy is an important clinical problem. Asthma presents with multiple phenotypes and endotypes (19). Identification of endotypes of asthma is the result of our increased understanding of the pathophysiology of the disease including the role that various immune pathways play in disease development and progression. Allergic asthma is the most common form of asthma, but allergic asthma is also a heterogeneous condition that could be associated with different endotypes (20). Serine proteases present in the airways have been associated with the pathogenesis of allergic asthma through their ability to activate PAR_2_.

## PAR_2_ in asthma pathogenesis

### PAR_2_ and allergic airway inflammation

The first evidence suggesting a role for PAR_2_ in asthma came from studies showing pro-inflammatory effects of PAR_2_-mediated airway epithelial cell activation (21, 22). More direct evidence came in 2002 when Schmidlin et al. showed that PAR_2_ knockout (KO) mice were protected from the development of eosinophilic airway inflammation and airway hyperresponsiveness (AHR) in response to ovalbumin (23). The latter observation has since been reproduced in murine models that utilize ovalbumin, but also various biologically relevant allergens (24–27).

**FIGURE 1:**
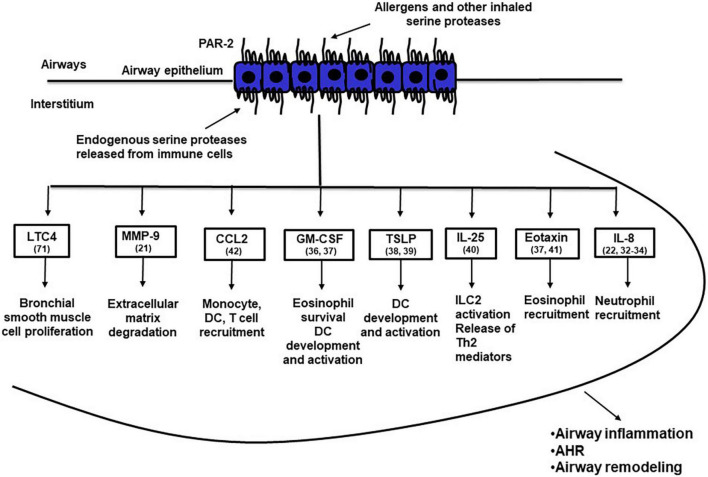
PAR-2 activated airway epithelial cells release a variety of inflammatory mediators that may induce airway inflammation, airway hyperresponsiveness and airway remodeling. The numbers in brackets after the name of specific inflammatory mediators indicate the reference of the main text where that mediator is discussed. AHR, airway hyperresponsiveness; CCL2, CC motif chemokine ligand 2; DC, dendritic cell; GM-CSF, granulocyte-macrophage colony-stimulating factor; ILC2, type 2 innate lymphoid cells; IL-25, interleukin 25; IL-8, interleukin 8; LTC4, leukotriene C4; MMP-9, matrix metalloproteinase 9; TSLP, thymic stromal lymphopoietin.

The airway epithelium, the first organ encountered by inhaled particles, pollutants and allergens, is viewed as an important immune organ aimed to protect the organism from environmental insults (28, 29), but is also involved in the pathogenesis of respiratory inflammatory diseases, including asthma (30, 31). PAR_2_-mediated activation of airway epithelial cells has been reported to release a number of factors that play important roles in asthma pathogenesis (Figure 1); these factors include remodeling proteases such as matrix metalloproteases (21), the neutrophil chemotactic factor IL-8 (22, 32–34), IL-6 (22, 35, 36), GM-CSF that affects multiple innate and adaptive immune cells (36, 37), the Th2 polarizing mediators TSLP (38, 39) and IL-25 (40) and various chemokines such as eotaxin (37, 41) and CCL-2 (42). These observations suggest that PAR_2_-mediated activation of the airway epithelium may release inflammatory mediators that polarize the immune response toward the Th2 phenotype and attract innate and adaptive immune cells to the airways (43).

**FIGURE 2:**
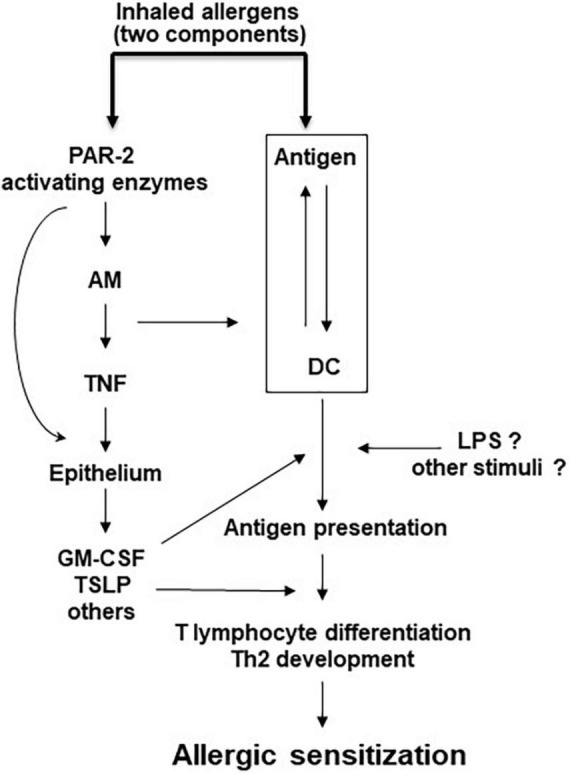
Model of PAR-2-mediated allergic sensitization. Allergic sensitization requires the recognition of an antigen by DC and also PAR-2 activation by an allergen with serine protease activity, or by an independent serine protease. Other stimuli may facilitate this process. AM, alveolar macrophages; DC, dendritic cells; GM-CSF, granulocyte macrophage colony stimulating factor; LPS, lipopolysaccharide; TNF, tumor necrosis factor; TSLP, thymic stromal lymphopoietin.

Development of allergic airway inflammation in humans and animal models can be divided into two steps; initial encounter with the antigen locally or systemically, leads to allergic sensitization with development of antigen-specific Th2 cells and production of IgE, while a subsequent exposure of a sensitized individual to the same antigen leads to the development of eosinophilic airway inflammation and AHR. PAR_2_ activation may participate in the development of allergic sensitization by inducing a deviation of the nasal/airway mucosa immune response against a foreign innocuous antigen from the default pathway of tolerance to allergic sensitization and production of antigen-specific IgE (44). This PAR_2_ effect exhibits striking similarities to the effects of TLR4 activation in the airways (45). We propose that in the airways PAR_2_ recognizes both internal and external “danger” signals, namely serine proteases released from inflammatory and other cells or inhaled through the air, respectively. PAR_2_ activation under these circumstances leads to activation of the innate and adaptive immune system through soluble mediators from the airway epithelium and to allergic sensitization (Figure 2). In addition to TNF (44), many other factors may also mediate, at least in part, the deviation of the immune system toward a Th2 phenotype and allergic sensitization following PAR_2_ activation, some of them factors released by the airway epithelium (Figure 1). In addition to the indirect effects of PAR_2_ activation on the adaptive immune system shown in Figure 2, endogenous and/or exogenous serine proteases may directly activate adaptive immune cells, since they also express PAR_2_ (46). Finally, in sensitized individuals, PAR_2_ activation during repeat exposures to sensitizing allergens results in release of inflammatory mediators important for the development of allergic airway inflammation, AHR and airway remodeling (14, 47, 48).

However, the role of PAR_2_ in the airways may be more complex that discussed so far. PAR_2_ activation causes relaxation of trachea preparations and protects from bronchoconstriction *in vivo* through the release of PGE_2_ from airway epithelial cells (49). Similarly, in a rabbit model of pollen allergy, PAR_2_ activation in the airways just prior to allergen challenge decreased allergen-mediated bronchoconstriction, eosinophil infiltration and AHR (50). These observations indicate that PAR_2_ may also have a protective effect against the development of signs and symptoms associated with asthma. The reasons for the potential dual effect of PAR_2_ activation have not been identified. We also do not know whether these “protective” effects of PAR_2_ activation can acutely or chronically antagonize the better studied pro-inflammatory effects. Finally, we don’t know if these protective effects would also be evident in human airways.

The vast majority of the *in vivo* data on the role of PAR_2_ in allergic inflammation come from animal studies. There is limited information on the pro-inflammatory potential of PAR_2_ in humans *in vivo*. PAR_2_-AP have been shown to induce inflammation when applied to humans intradermally (51), but these peptides have not been administered to humans through any other routes.

PAR_2_ may also affect allergic airway inflammation through its expression on a variety of immune cells. Both monocytes and macrophages express PAR_2_ (52) and its expression is altered in airway inflammatory conditions (53). PAR_2_ activation leads to cytokine production from monocytes and macrophages (48, 54, 55), affects macrophage differentiation (56, 57) and has antiviral effects. *In vitro* differentiated DC do not express PAR_2_ (52), but PAR_2_ is needed for their normal maturation (58). PAR_2_ contributes to DC antigen uptake and facilitates the presence of mature DC in draining lymph nodes *in vivo*, but in these case it is not clear if the effects are direct (44, 59). Direct PAR_2_ activation on naive T cells by proteases may induce IL-4 release and lead to allergic inflammation (46). PAR_2_ activation induces various inflammatory, but also antiviral pathways in neutrophils (60–63). Finally, eosinophils may also express PAR_2_, but the role of PAR_2_ in eosinophil functions is controversial (64–66).

### PAR_2_ and airway remodeling

Airway smooth muscle cells also play an important role in asthma pathogenesis (67). In addition, asthma, especially severe disease, is characterized by airway remodeling that includes airway smooth muscle hyperplasia and hypertrophy and fibrosis (30, 68). Two studies using house dust mite (HDM), showed that allergen proteases also induce proliferation of asthmatic bronchial smooth muscle cells through PAR_2_-dependent mechanisms (69, 70). These observations suggest that PAR_2_-mediated smooth muscle activation, either directly or indirectly through LTC_4_ released from epithelial cells (71), may contribute to the smooth muscle hypertrophy and/or hyperplasia seen in patients with asthma.

There are *in vivo* observations that PAR_2_ activation is involved in fibrosis, but these come primarily from fibroproliferative lung diseases such as idiopathic pulmonary fibrosis. A murine study showed that PAR_2_ contributes to the development of pulmonary fibrosis, while targeting PAR_2_ affords protection from bleomycin-induced fibrosis (72). Another study showed that mast cell tryptase induces lung fibroblast proliferation via PAR_2_-activation (73), suggesting that activated mast cells may induce fibrotic changes in asthma through PAR_2_ activation.

### Regulation of PAR_2_ expression

If PAR_2_ is important for development of allergic sensitization and inflammation, then interfering with its expression or activation may be a viable approach for prevention and/or treatment of allergic diseases. However, triggers relevant to asthma may upregulate PAR_2_ expression in the airways, which in turn may exacerbate allergic airway inflammation. PAR_2_ expression is increased on the airway epithelium of asthmatic individuals (74) and on the nasal mucosa epithelium of patients with allergic rhinitis (75, 76). Various inflammatory mediators upregulate PAR_2_ expression on endothelial cells (77, 78), mast cells (79, 80) and other cells (81–83), and the same may be true for airway epithelial cells. Also, cockroach (34), HDM (84), and mold (33) allergen extracts upregulate PAR_2_ expression on airway epithelial cells, possibly through proteases contained within the extracts. In addition, inflammatory stress, which is present in asthmatic airways, may regulate PAR_2_ expression in the lung through hypoxia, as has been shown to do in endothelial cells (85).

Bronchial smooth muscle cells from asthmatic individuals maintain higher PAR_2_ mRNA and protein expression than cells from normal individuals after *ex vivo* culture (86), suggesting the possibility that epigenetic changes due to the chronic inflammation in the airways may affect PAR_2_ expression. We recently showed that insulin regulates PAR_2_ expression in primary human airway epithelial cells through the FOXO1 transcription factor (87), which may indicate that insulin resistance, often associated with asthma (88, 89), may be associated with alterations of PAR_2_ expression. Finally, genetic factors regulating PAR_2_ expression cannot be excluded as PAR_2_ SNPs have been shown to increase mRNA stability and increase expression of PAR_2_ in PBMCs (90) and synovial tissue (91).

Prevention of PAR_2_ activation by allergens or endogenous proteases may also have therapeutic benefits in asthma. Unfortunately, development of small molecule inhibitors has been problematic, and only recently such PAR_2_ inhibitors are being described (92). A monoclonal humanized antibody has also been described, but has not been tested whether it is functional *in vivo* (93). Another antibody (MEDI 0618) is undergoing phase I evaluation and results may be available soon.^1^

## PAR_2_ expression as an asthma biomarker

Personalized medicine offers promise for improved diagnosis and treatment for inflammatory diseases including lung diseases (94, 95), but in asthma the lack of easily obtainable biomarkers to identify specific phenotypes and/or endotypes (96), limits the applicability of this approach. Many biomarkers have been tested and they all have their advantages and disadvantages (97). Identification of patients with severe asthma is an important clinical question, since these individuals require more intense treatment and close follow up to avoid asthma morbidity. Biomarkers that could identify those individuals and predict their response to therapy are in great demand (98).

Over the last few years our laboratory has been studying the utility of PAR_2_ expression as a biomarkers of asthma severity and control. Cells obtained through induced sputum would be ideal for these studies, but they are not easily accessible, except in specialized centers. Therefore, we have focused on peripheral blood cells. We validated that a subset of peripheral blood monocytes express surface PAR_2_, as has been shown before (48), but our more interesting observation was that cell surface PAR_2_ expression on peripheral blood intermediate monocytes (IMMo) correlated with disease severity (99). In particular, patients with severe asthma had higher% of IMMo expressing PAR_2_ and higher total number of PAR_2_-expressing IMMo in their peripheral blood compared to subjects with mild/moderate disease. Other cells, including eosinophils, neutrophils, and CD4^+^ lymphocytes, showed low PAR_2_ expression and no differences in expression between the two populations with different asthma severity, as there was also no difference between the two groups in PAR_2_ expression in classical monocytes. Our data showed that PAR_2_ expression on IMMo was an excellent marker to discriminate between subjects with severe and those with mild/moderate asthma. PAR_2_ expression on monocytes of patients with rheumatoid arthritis (100), granulomatosis with polyangiitis (101) and primary antiphospholipid syndrome (102) also correlates with disease activity. However, asthma is the first inflammatory condition where changes in PAR_2_ expression in a specific monocyte subgroup are associated with disease severity.

In addition, the% of PAR_2_-expressing IMMo in peripheral blood correlated with the dose of inhaled steroids prescribed to these subjects and was higher in subjects that had experienced at least one exacerbation over the last year. Unfortunately, we did not have detailed information on the proximity, total number and severity of exacerbations to understand whether subjects with a recent exacerbation were those with an exacerbation prone phenotype, a phenotype that has been shown to have prognostic significance for severe asthma.

It is also interesting that in the population with a recent asthma exacerbation PAR_2_ mRNA expression also correlated with the numbers of Th2 cells in the peripheral blood, indicating that PAR_2_ expression may also be associated with T2 inflammation, although the mechanisms leading to this association are not clear. It is interesting that PAR_2_-mediated activation of macrophages induces IL-4 secretion (103), which might contribute to T2 environment in peripheral tissues and may even support the development of allergic sensitization, an effect that follows PAR_2_ activation in murine studies (26, 44). It is also possible that the same triggers that lead to T2 disease or factors present in subjects with T2 diseases, are those that upregulate PAR_2_ on monocytes. To this effect we have evidence that LPS, which through TLR4 activation can facilitate allergic sensitization (45) or CCR5 that is increased in the airways of subjects with asthma (104, 105), can both upregulate PAR_2_ on human IMMo *in vitro* (106).

From our data comparing PAR_2_ expression in IMMo between subjects with severe and mild/moderate asthma, it is not clear whether PAR_2_ upregulation on the surface of IMMo depends on asthma severity or the presence of uncontrolled inflammation that can be present in severe disease. Two studies shed some light to this question. In a recent study we showed that PAR_2_ expression on the surface of IMMo is increased during an asthma exacerbation (107). In this study we showed that PAR_2_ expression on peripheral blood IMMo is higher in subjects presenting to the Emergency Department (ED) with an exacerbations compared to subjects with stable disease. PAR_2_ expression comes down to levels present in subjects with stable disease 2 weeks after the ED presentation and after the exacerbation has been treated. It is possible that increased inflammation in the days leading to an exacerbation is the reason for increased PAR_2_ expression. Increased systemic inflammation may lead to increased PAR_2_ expression on IMMo, as suggested by our results using a human allergy challenge model. In that case, inhalation allergen challenge induced an early (6 h) increase in PAR_2_ expression on peripheral blood IMMo that was sustained at 24 h (107). It would be interesting to know whether the same changes in PAR_2_ expression are also seen in inflammatory cells in the airways. Studies are underway in our laboratory to understand whether PAR_2_ expression increases in induced sputum and/or BAL cells after an allergen challenge and also in subjects with uncontrolled versus controlled asthma.

One of the requirements for PAR_2_ expression on peripheral blood IMMo to be used as a biomarker is that this value is stable and reproducible during the course of stable disease. Our data however, show that this may not be the case (108). We recruited 20 stable asthmatics and repeated the evaluation of PAR_2_ expression on peripheral blood IMMo every 3 months for a year. We found that even though the% of IMMo expressing PAR_2_ was stable in the whole population, there were differences in expression for specific individuals that could not be explained with the available clinical data. In this study we had no subjects that experienced an asthma exacerbation. It is possible that changes preceding exacerbations will be greater that the fluctuation of values during a stable course of the disease and therefore, this value may be useful as predictive biomarker for asthma exacerbations.

Being able to evaluate the activation state of the receptor, instead of its presence on the cell surface, may be a more accurate approach to evaluate the activity of this inflammatory pathway and may also function as a biomarker that could be used in asthma. A recent study showed that the small peptide liberated from the receptor when it is cleaved by activating serine proteases can be detected in human serum and its levels increase in patients with rheumatoid arthritis and responds to treatment of the disease with specific biologics (109). It will be interesting to test whether the levels of this peptide also change in asthma and whether it may be used as another biomarker for asthma severity and/or control.

## Conclusion

Asthma is an inflammatory disease of the airways. Even though we can treat successfully the disease in the vast majority of subjects with mild or moderate asthma, we still are not able to fully address the needs of patients with severe disease. In addition, we know that exacerbations, especially severe exacerbations requiring urgent care, can happen at any point even in patients with mild disease and reliable biomarkers to predict such events are missing.

Our current knowledge on the potential role of PAR_2_ in allergic asthma, indicates that markers of activation of PAR_2_-related pathways may be candidates for biomarkers. Our current observations may allow the development of new hypotheses regarding potential biomarkers of asthma severity or impending exacerbations, that could be tested in future studies.

## Author contributions

VG, NS, and HV were responsible for study design and authored the final draft of review article. All authors read and approved the final review article.

## Footnotes

1
https://clinicaltrials.gov/ct2/show/NCT04198558

## Abbreviations

AM: Alveolar macrophage
AP: Activating peptide
AHR: Airway hyper responsiveness
BAL: Bronchial alveolar lavage
CCR5: C-C chemokine receptor type 5
DC: Dendritic cell
ED: Emergency department
FOXO1: Forkhead Box O1
GM-CSF: Granulocyte macrophage colony stimulating factor
HDM: House dust mite
IL: Interleukin
IgE: Immunoglobulin E
IMMo: Intermediate monocytes
LPS: Lipopolysaccharides
LTC_4_: Leukotriene C4
PAR: Protease activated receptor
SNP: Single nucleotide polymorphism
TL: Tethered ligand
TLR4: Toll like receptor 4
TNF: Tumor necrosis factor
TSLP: Thymic stromal lymphopoietin.
